# Radiographic characterization of OPLL progression in patients receiving laminoplasty with a minimum of two-years follow-up

**DOI:** 10.1007/s10143-024-02735-z

**Published:** 2024-08-29

**Authors:** Wai Kiu Thomas Liu, Keira Ho Yuet Siu, Jason Pui-Yin Cheung, Graham Ka-Hon Shea

**Affiliations:** Department of Orthopaedics and Traumatology, Li Ka Shing Faculty of Medicine, The University of Hong Kong, Pok Fu Lam, China

**Keywords:** Ossification of the posterior longitudinal ligament, Cervical myelopathy, Laminoplasty

## Abstract

Ossification of the posterior longitudinal ligament (OPLL) is a common cause of degenerative cervical myelopathy (DCM) in Asian populations. Characterization of OPLL progression following laminoplasty remains limited in the literature. 29 patients with OPLL received cervical laminoplasty and a minimum of 2-years follow-up. Clinical and radiological surveillance occurred at 3-months, 6-months, 12-months post-op and then at yearly intervals. Transverse (anteroposterior) diameter and sagittal length of OPLL in relation to their cervical vertebral level of localisation was assessed upon immediate post-op radiographs compared to those obtained at subsequent follow-up. OPLL progression was defined as an increase in transverse dimensions and/or length by ≥ 2 mm. The average period of clinical follow-up was 6.7 ± 3.3 years. Upon latest follow-up, 79% of patients demonstrated at least 2 mm of transverse or longitudinal progression of OPLL. This corresponded to 2-years and 5-year progression rates of 54% and 71% respectively. OPLL located over C5 demonstrated the greatest transverse progression rate at (0.24 ± 0.34 mm / year). The mean overall longitudinal progression rate was 1.61 ± 2.06 mm / year. No patients experienced neurological decline resulting from OPLL progression requiring revision decompression during the period of post-operative observation. Characterizing transverse and longitudinal progression by cervical level via radiographs has implications in surgical planning for OPLL and should be consolidated upon post-operative CT/MRI scans as well as larger sample sizes.

## Background

Ossification of the posterior longitudinal ligament (OPLL) is a cause of degenerative cervical myelopathy (DCM) that is particularly common in East Asian countries, with a population prevalence of 0.4 to 3.0% [1, 2]. OPLL most often affects the C4 to C6 cervical levels, whilst classification is according to radiographic findings and includes continuous, mixed, segmental, and localized subtypes [1]. Continuous and mixed OPLL have been shown to be associated with higher rates of progression [3, 4]. The exact pathogenesis of OPLL is unclear but suggested to be secondary to both genetic and environmental factors [5]. Single-nucleotide polymorphism (SNP) of collagen genes have been implicated in pathogenesis [6, 7] whilst dysfunction of human nucleotide pyrophosphatase (NPPS) is proposed to exacerbate tissue calcification [8]. In addition to genetic factors, mechanical stress is a risk factor [9].

The current standard of care in the treatment of OPLL with neurological impairment is to offer surgical decompression. Direct anterior decompression may be more difficult to achieve when the ossified segment lies posterior to the vertebral body thereby requiring a corpectomy, and as adhesions between the OPLL and ventral dura increases the risk of dural tear and CSF leak [10, 11]. Therefore, when cervical spine alignment is favourable [12], indirect decompression via a posterior approach is may be preferred particularly in multilevel and/or congenital canal stenosis. For both anterior and posterior approaches, there is the concern that OPLL progression following surgery could lead to recurrence of compression followed by neurological deterioration.

With regards to posterior cervical surgery for OPLL, a recent meta-analysis of laminoplasty in comparison to laminectomy and fusion demonstrated similar neurological outcomes and range of motion after surgery. Shorter operation time and less blood loss was demonstrable following laminoplasty whilst cervical lordotic angle was higher following fusion [13]. It has been reported that up to 70% of patients exhibit progression of OPLL following laminoplasty due to sparing of cervical motion [14]. Nevertheless, recommending laminectomy and fusion when OPLL is treated via a posterior approach remains controversial as some authors have conversely demonstrate increased ossification following fusion [15], the clinical significance of post-operative OPLL progression remains conflicting [3, 4], and outcomes at 2-years following laminoplasty remain good in spite of OPLL progression [16]. An additional issue that remains unknown is how OPLL progression differs by cervical level, which has implications on surgical approach, planning, and execution. To address these issues, this study investigated the rates of radiological OPLL progression according to cervical level in patients receiving laminoplasty, with an average post-operative follow-up period exceeding 6-years.

## Methods

### Patient recruitment and follow-up

**Fig. 1:**
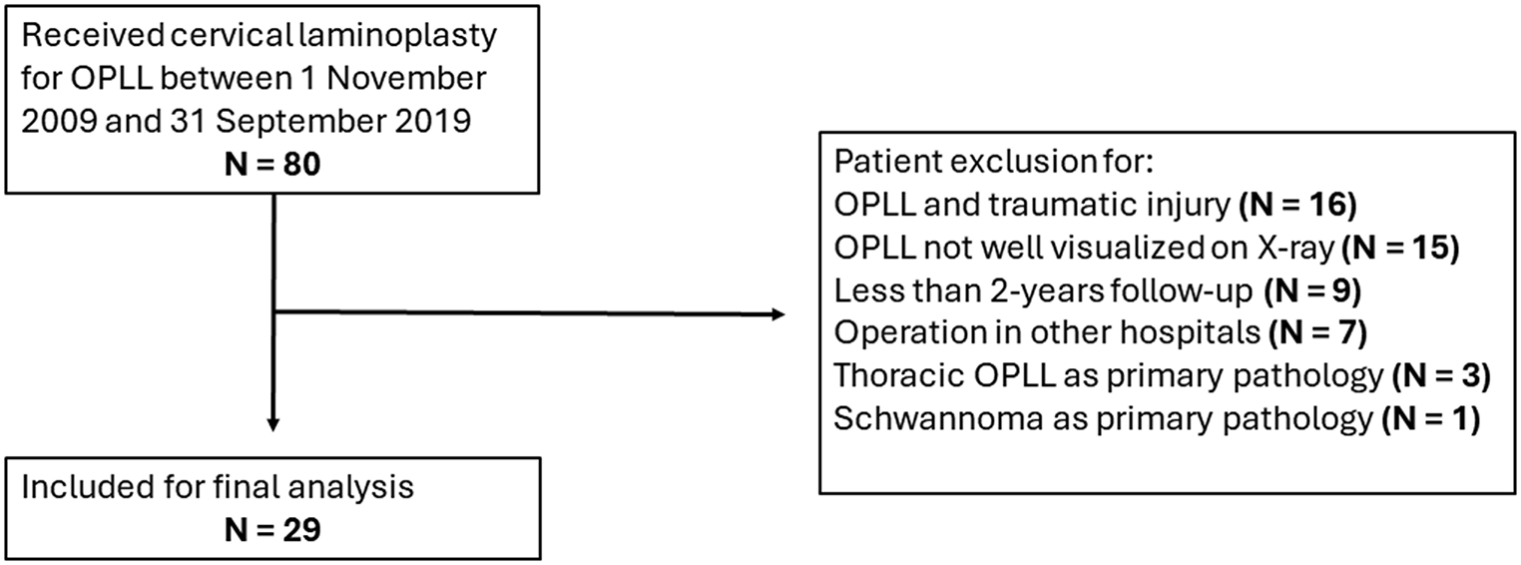
Flowchart for patient recruitment

This study was approved by the Institutional Review Board (IRB) of our institution and conducted according to the principles detailed in the Preferred Reporting Of CasE Series in Surgery (PROCESS) Guidelines. We identified patients with DCM and radiological evidence of cervical canal narrowing due to OPLL who had undergone cervical laminoplasty between 1 November 2009 and 31 September 2019 at an academic spinal unit. At our centre, we favour laminoplasty for indirect decompression in patients with cervical OPLL and a stable lordotic cervical spine. Our rationale for favouring laminoplasty over laminectomy and posterior fusion is due to its motion-sparing nature and non-inferior clinical outcomes together with lower risk of C5 palsy [17–19]. Open door laminoplasties were performed as previously described, with a full-thickness trough made at the junction of the laminae and lateral mass on one side, and a partial-thickness trough over the contralateral side to allow for lamina opening [20]. Upon discharge, patients were assessed at 2-weeks to review the wound condition, then at 3-months, 6-months, 12-months and yearly intervals for clinical and radiologically monitoring. We excluded patients suffering from DCM secondary to other causes, patients without radiological evidence of OPLL upon lateral cervical X-rays, patients having received previous cervical spinal operations, patients with neurological deterioration associated with traumatic cervical injury, and patients with less than 2 years of clinical and radiological follow-up. 80 patients with an operative diagnosis of ossified posterior longitudinal ligament who underwent cervical laminoplasty in the abovementioned period were identified using the Clinical Data Analysis and Reporting System of the Hospital Authority, Hong Kong. A flowchart for patient recruitment, resulting in 29 patients included for analysis, is shown in Fig. 1.

Demographic data, including age at operation, gender, details of the laminoplasty procedure, pre- and post-operative neurological function (modified JOA score) [21] and occurrence of any surgical complications (infection, hardware malposition, neurological deterioration) were retrieved from electronic medical records. Lateral cervical x-rays from pre- and post-operative periods, as well as pre-op cervical MRIs, were retrieved from the hospital picture archiving and communication system (PACS).

### Measurement of OPLL progression and cervical parameters

**Fig. 2:**
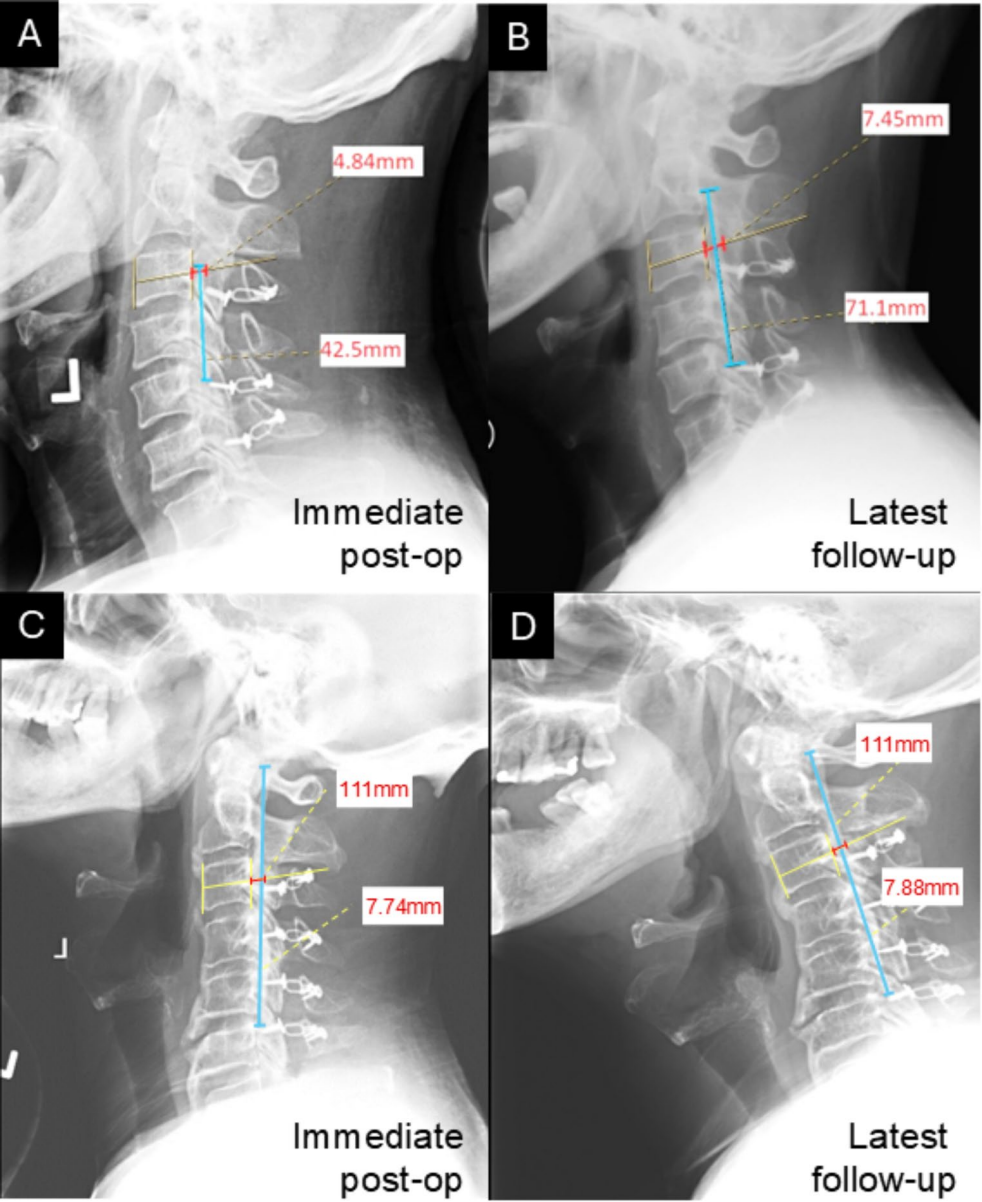
Radiological measurement of OPLL progression. Lateral cervical X-rays demonstrating the measurement of transverse (red; at C3 level) and longitudinal (blue) extent of OPLL immediately post-op and that taken at latest follow-up in representative cases with and without radiological progression. Panels **A** and **B** illustrate a case of progression, whilst panels **C** and **D** illustrate a case of non-progression after more than 5 years of follow-up

Cervical radiographs were taken at the radiology department of a single institution in a standardized manner. From lateral radiographs, the levels affected and subtype of OPLL involvement was determined [1]. The transverse thickness (anteroposterior diameter) of OPLL at each cervical level was measured on neutral (i.e. not flexion or extension view) lateral cervical X-rays by drawing a line from the midpoint of the anterior border of the vertebra to the midpoint of the posterior border of the vertebra, and by extending this line to the posterior-most extent of OPLL visible radiologically, for every affected cervical vertebral level (Fig. 2). Specifically, the transverse thickness of OPLL at the affected level was the distance from the midpoint of the posterior vertebral border to the posterior-most extent of OPLL visible radiologically. OPLL sagittal length was measured from its cranial border to its caudal-most extent. The corresponding mid-sagittal diameter of the spinal canal posterior to the OPLL was measured from pre-op MRI scans. Measurements at 2-year and 5-year post-op as well as latest follow-up was compared with X-rays taken immediately after the operation. OPLL progression was defined as an increase in transverse thickness or length by ≥ 2 mm in accordance with previous studies [1, 3, 4, 22–24]. Progression rate was calculated by measuring the difference between the transverse thickness or length of the OPLL following a defined post-operative period and dividing by the duration between radiographs. Cervical sagittal alignment was measured on neutral lateral X-rays between the inferior endplate of C2 and the inferior endplate of C7. If the inferior endplate of C7 was not well visualised, the inferior endplate of C6 was referenced instead. mJOA scores were retrieved from either electronic / physical doctor notes or occupational therapy reports at both the immediate pre-op period as well as upon post-op follow-up.

### Statistical analysis

Analysis of variance (ANOVA) was utilised to compare the progression rates at different cervical levels and at different time points from surgery. The intraclass correlation coefficient was calculated from transverse and longitudinal OPLL measurements taken by two observers to assess inter-observer reliability. To compare the progression rate of individual cervical levels, independent t-tests were conducted with a Bonferroni correction applied to account for multiple comparisons. Paired t-test was utilized to analyse the difference between the annual progression rate of the first two years after the operation with that of the subsequent period, in patients with assessment at three or more years after surgery. Chi squared testing was utilized to compare categorical variables. Reported numbers represent mean ± standard deviation unless otherwise stated. Statistical analyses were conducted using SPSS (version 27.0, IBM, Armonk, New York, USA).

## Results

### Demographic and post-operative details of study cohort

A total of 29 patients consisting of 19 males and 10 females were recruited for analysis. The demographics of subjects with and without transverse OPLL progression are shown in Table 1. The average age at operation was 61.3 ± 6.7 years old (range of 48–76). Body mass index at the time of operation was 25.0 ± 3.2. A total of 24 of 29 patients had X-rays available at exactly 24-months after the operation, whilst 18 of 29 patients had X-rays at 5-years or more after the operation. 18 patients were classified as having severe DCM (mJOA < 12) according to the preoperative mJOA scores, whilst the remaining 11 patients had moderate DCM (mJOA = 12–14). In 20 of the patients receiving laminoplasty, the hinge was held open by plates, whilst sutures were utilized in the remaining 9 cases. The mean number of cervical levels receiving laminoplasty was 4.2 ± 0.5 (range, 3–5). Patients most often received four-level laminoplasties (C3-C6, 21 patients) and five-level laminoplasties (C3-C7, 7 patients).

**Table 1:** Clinical and radiographic details in relation to transverse progression

	Overall	Transverse progression	No transverse progression	*p*-value
Patient number	29	13 (45%)	16 (55%)	N.A.
Gender (M/F)	19/10	9/4	10/6	0.705
Mean age	61.3 ± 6.7	60.3 ± 6.4	62.1 ± 7.2	0.482
BMI (kg/m^2^)	25.0 ± 3.2	25.9 ± 3.2	24.0 ± 3.0	0.159
Clinical follow-up duration (years)	6.7 ± 3.3	7.5 ± 3.1	6.1 ± 3.4	0.247
Radiological follow-up (years)	6.0 ± 3.1	6.6 ± 3.1	6.1 ± 3.2	0.340
Preoperative mJOA score	9.5 ± 3.5	8.8 ± 4.4	9.1 ± 3.1	0.601
Severity of DCM according to mJOA score	Moderate: 11/29 (37.9%) Severe: 18/29 (62.1%)	Moderate: 5/13 (38.5%) Severe: 8/13 (61.5%)	Moderate: 6/16 (37.5%) Severe: 10/16 (62.5%)	0.958
OPLL subtype	I: 17 (59%) II: 2 (7%) III: 7 (24%) IV: 3 (10%)	I: 7 (54%) II: 1 (8%) III: 4 (31%) IV: 1 (8%)	I: 10 (63%) II: 1 (6%) III: 3 (19%) IV: 2 (13%)	0.873
Pre-op cervical lordosis	9.1 ± 9.0^o^	6.3 ± 10.4^o^	11.4 ± 7.2^o^	0.129
Transverse OPLL diameter over thickest region	4.00 ± 1.59 mm	3.57 ± 1.45 m	4.35 ± 1.65 mm	0.135
Laminoplasty by plate	20/29 (69%)	9/13 (69%)	11/16 (69%)	0.978
Levels of laminoplasty	Five-level: 7 (24%) Four-level: 21 (72%) Three-level: 1 (3%)	Five-level: 3 (23%) Four-level: 10 (77%)	Five-level: 4 (25%) Four-level: 11 (69%) Three-level: 1 (6%)	0.641

BMI: body mass index, DCM: degenerative cervical myelopathy; mJOA score: modified Japanese Orthopaedic Association score, OPLL: ossified posterior longitudinal ligament. OPLL subtype: I = continuous, II = segmental, III = mixed, IV = localized

### Prevalence of OPLL by cervical level and type

Radiological assessment by OPLL by subtype revealed that 17 were continuous, two were segmental, seven were mixed type (continuous and segmental) and three were localised. The mean number of cervical levels with OPLL involvement was 4.1 ± 1.3 (range, 2–7). The AP diameter of OPLL at the thickest affected region was 4.00 ± 1.59 mm. The cervical levels affected by OPLL are summarized in Table 2, with C4 (29/29 patients), C3 (26/29) and C5 (23/29) levels most often involved. The mean transverse diameters upon immediate post-operative X-rays demonstrated that OPLL was thickest at C3, followed by C4 and C2. Sagittal dimensions of the spinal canal posterior to the OPLL were narrowest over C3, followed by C4, and C5, at 6.63 ± 1.63 mm, 6.70 ± 1.59 mm, and 7.27 ± 1.46 mm respectively.

**Table 2:** Prevalence and size of OPLL by cervical level

Cervical level affected by OPLL	Number of patients	Transverse diameter of OPLL (± SEM)	Spinal canal diameter posterior to OPLL (± SEM)
C1	9	2.46 ± 0.70 mm	11.65 ± 1.99 mm
C2	21	4.57 ± 0.56 mm	8.67 ± 1.76 mm
C3	26	6.20 ± 0.47 mm	6.63 ± 1.63 mm
C4	29	5.25 ± 0.35 mm	6.70 ± 1.59 mm
C5	23	4.01 ± 0.47 mm	7.27 ± 1.46 mm
C6	10	1.82 ± 0.50 mm	8.49 ± 1.50 mm
C7	1	0.53 ± 0.53 mm	10.0 ± 1.96 mm

SEM: Standard error of the mean

### Overall OPLL progression

Amongst the entire cohort, 79% (23/29) of patients demonstrated at least 2 mm of transverse or longitudinal progression of OPLL at latest follow up. At 2-years and 5-years post-op, these corresponded to 54% (13/24) and 71% (10/14) of the cohort (Table 3).

**Table 3:** 2-year and 5-year overall, transverse, and longitudinal OPLL progression rates

	Overall OPLL progression (transverse or longitudinal)	Transverse OPLL progression	Longitudinal OPLL progression	*p*-value (transverse vs. longitudinal)
2-year	54% (13/24)	17% (4/24)	50% (12/24)	0.014
5-year	71% (10/14)	21% (3/14)	71% (10/14)	0.008
Latest follow-up	79% (23/29)	45% (13/29)	69% (20/29)	0.063

### Transverse OPLL progression

The prevalence of transverse OPLL progression at latest follow-up was 45% (13/29). Mean transverse progression rate at latest follow-up regardless of cervical level was 0.14 ± 0.23 mm / year (range 0–1.34 mm). The mean transverse progression rate in the first two years after the operation was compared to the mean transverse progression rate in subsequent years (4.8 ± 2.7 years; range 1.0–10.7). Transverse progression rate in the first two years after laminoplasty was 0.20 ± 0.48 mm/year (range 0–3.40), while that in the subsequent 4.8 years this fell slightly (*p* = 0.643) to 0.17 ± 0.36 mm/year (range 0–2.01).

**Fig. 3:**
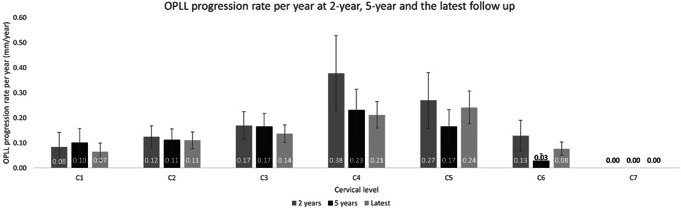
Transverse OPLL progression by cervical level. Average transverse OPLL progression (mm/year) at different cervical levels, by comparing the initial post-operative X-ray to the X-ray taken at 2-year, 5-year and the latest follow-up of each patient. The error bars represent the standard errors of the mean

With regards to cervical level, C5 demonstrated the greatest transverse progression rate at latest follow-up (0.24 ± 0.34 mm/year) followed by the C4 level (0.21 ± 0.28 mm/year). When comparing the transverse progression rate of each cervical level, statistically significant differences were found in comparison of C4, C5 and C6 with C7 (*p* < 0.01). Averaged rates of transverse progression at 2-year, 5-years, and latest follow-up for each cervical level are illustrated in Fig. 3. The intraclass correlation coefficient for inter-observer measurements of transverse OPLL dimensions upon lateral cervical X-rays was 0.826 (95% CI: 0.789–0.856).

### Longitudinal OPLL progression

69% (20/29) of patients demonstrated longitudinal progression upon their latest X-rays. This corresponded to 50% (12/24) and 71% (10/14) of patients at 2-year and 5-year post-op respectively. The mean longitudinal progression rate at latest follow-up was 1.61 ± 2.06 mm / year (range 0–8.64). The annual longitudinal progression rate in the first two years after the operation was compared to the annual longitudinal progression rate in subsequent years (4.8 ± 2.7 years; range 1.0–10.7) in 17 patients. The annual longitudinal progression rate in the first two years was 2.19 ± 2.75 mm / year (range 0–8.38) decreased to 1.05 ± 1.29 mm / year (range 0–4.96) in the subsequent period which was statistically significant (*p* = 0.042). The intraclass correlation coefficient for inter-observer measurements of sagittal OPLL dimensions upon lateral cervical X-rays was 0.754 (95% CI: 0.617–0.847).

### Sagittal alignment

Before the operation, 22 patients were K-line positive, while 7 were K-line negative. Patients with a negative K-line prior to operation demonstrated a lordotic alignment upon extension films. As of the most recent radiological assessment, 16 patients were K-line positive and 13 were K-line negative. The mean pre-operative cervical sagittal alignment was 9.1 ± 9.0^o^ of lordosis as compared to 4.1 ± 8.3^o^ at latest follow-up which was a statistically significant decrease (*p* < 0.001).

### Complications and neurological survivorship

One patient was noted to have deterioration in left side motor power immediate post-operation but demonstrated spontaneous neurological improvement. He also received debridement for wound infection at 2-weeks post-op. Amongst the overall cohort, preop mJOA was 9.13 ± 3.66 compared to 13.21 ± 2.34 upon peak post-op recovery (*p* < 0.01). The overall mean improvement in mJOA after laminoplasty was 4.33 ± 3.51, and the neurological recovery rate (NRR) was 53.9 ± 28.6%. The mean mJOA increment of patients with transverse progression was 4.33 ± 5.39, while that of those without transverse progression was 4.33 ± 3.11 (*p* = 1.000). No patients required re-operation for neurological deterioration at latest follow-up.

## Discussion

This study examined the progression rate of cervical OPLL following laminoplasty. Our results showed that longitudinal progression occurred more frequently than transverse progression. Reassuringly, none of the patients required revision surgery for neurological deterioration after an average follow-up period exceeding 6-years. To the best of our knowledge, this work represents the first attempt to characterize OPLL progression rate by cervical level affected. Our results are of relevance to surgical planning and patient counselling.

A recent systematic review revealed that cervical laminectomy and fusion resulted in an OPLL progression rate of only 22.7% as compared to 68.8% following cervical laminoplasty [25], whilst offering similar neurological outcomes [23, 24, 26–29]. Our results were consistent with this trend, yet laminoplasties remain as a good treatment option in patients with OPLL and a favourable cervical alignment given the absence of neurological deterioration upon mid-term follow-up. Our findings on greatest OPLL progression and canal narrowing at C3-C5 has ramifications on both the execution of laminoplasties, as well as the choice of surgical technique, particularly in a younger patient population. Laminoplasties should be performed with greater hinge opening at these at-risk levels and care to preserve and repair the soft tissues to facilitate greater float back. The opened lamina hinge must be secured with a technique that prevents ‘spring back’, which is common when sutures are used. With regards to choice of surgical technique, younger and fitter patients with sizeable OPLL at C3- C5 and marginal clearance of the K-line would benefit more from direct anterior decompression or posterior instrumented fusion, in view of anticipated radiological progression and associated risk of neurological decline over the long-term. Nevertheless, it remains essential that evidence for post-laminoplasty neurological deterioration due to accelerated OPLL progression at C3-5 is substantiated upon longer follow-up duration.

Our findings for overall progression at 2-years following laminoplasty (54%) corresponded to the literature, which ranged from 38.5 to 56.5% within a similar observational period [3, 30]. Similarly, our reported prevalence of progression at 5-years post-op (71%) was comparable to published studies reporting a progression rate of 50–75% [22, 31, 32]. Cervical laminoplasty is a motion-sparing surgery and retention of intervertebral motion has been attributed as a biomechanical factor causing OPLL progression [33]. The preponderance for OPLL development [1] and progression at C4 and C5 may be attributed to the relatively greater range of motion at this region compared to the rest of the subaxial cervical spine. Specifically, the flexion and extension range of C4/5 and C5/6 has been documented respectively to be 16–23^o^ and 15–28^o^ [34–37]. Our findings also revealed that longitudinal OPLL progression was more prevalent than transverse progression. Mean longitudinal progression rate (1.61 mm/year) was compatible to the range documented in prior studies (0.7 to 2.7 mm/year) [14, 24, 38, 39]. Upon further characterization with interval CTs, longitudinal progression may indicate a need for prophylactic decompression of cervical levels with relative stenosis that are yet to be affected by OPLL.

mJOA scoring is widely considered the gold standard to assess for DCM severity and post-operative recovery rates and exhibits high inter-rater reliability [40]. Reassuringly, recovery trajectories as evidenced by ΔmJOA was unaffected by OPLL progression. Whilst these findings indicate the general effectiveness of cervical laminoplasty, a caveat concerned the modest cohort size which may have resulted in statistical tests being underpowered, precluding subgroup analysis of neurological outcomes by OPLL subtyping, and by myelopathy severity, as most patients possessed mJOA scores between ‘moderate’ and ‘severe’ thresholds.

### Limitations

One of the main limitations of this study was that measurements were based on X-rays alone. Accuracy of the measurements could be affected by the quality and the projection of radiographs as well as the degree of calcification. Nevertheless, we demonstrated substantial agreement in the intra-class correlation coefficient between readers. CT scans were not routinely ordered for post-operative monitoring yet would have offered superior accuracy in determining OPLL dimensions. Post-operative outcomes would have been more comprehensive upon a longer duration of observation, especially for axial progression and recurrence of cord compression, and with the addition of patient-reported outcome measures [41]. Last but not least, a larger cohort size would be essential to further delineate the relationship between radiological progression of cervical OPLL and neurological recovery, and to allow for subgroup analysis. Whilst our study highlights post-operative radiological OPLL progression, the necessity for studies on population-wide OPLL detection followed by surveillance may be essential for East Asian locales where this disease entity is prevalent [42] and is tragically often only diagnosed following traumatic spinal cord injury.

## Conclusion

Despite radiological progression following laminoplasty, neurological outcomes and survivorship was excellent within the observation period. The rate of progression was greater longitudinally than transversely. Transverse progression of C5 OPLL was highest in comparison to other cervical levels. Our findings are of relevance to the selection of surgical procedure and execution of laminoplasties to minimize the risk of future cervical cord compression. Follow-up studies should be supplemented with larger patient numbers, longer follow-up duration and serial CT/MRI evaluation.

## Data Availability

No datasets were generated or analysed during the current study.
